# Aromatic Terpenes and Their Biosynthesis in *Dendrobium*, and Conjecture on the Botanical Perfumer Mechanism

**DOI:** 10.3390/cimb45070337

**Published:** 2023-06-25

**Authors:** Zhihui Du, Xiyu Yang, Shuting Zhou, Yuxuan Jin, Weize Wang, Kuaifei Xia, Zhilin Chen

**Affiliations:** 1 Guizhou Horticulture Institute, Guizhou Academy of Agricultural Sciences, Guiyang 550000, China; dzh8928088@163.com (Z.D.); xiyuyang.xy@outlook.com (X.Y.); jinyuxuan@whu.edu.cn (Y.J.); wangweize_gz@163.com (W.W.); 2 Natural Products Research Center of Guizhou Province, Guiyang 550000, China; zstaipt0117@163.com; 3 Guangdong Provincial Key Laboratory of Applied Botany, South China Botanical Garden, Chinese Academy of Sciences, Guangzhou 510650, China

**Keywords:** *Dendrobium*, floral scent, aromatic characteristics, terpene synthase, botanical perfumer mechanism

## Abstract

This review presents a systematic analysis of the studies on volatiles in *Dendrobium*. Among the various components, aromatic terpenes are a crucial component in the development of the aromatic characteristics of *Dendrobium* and other plants. Recent advancements in detection and sequencing technology have resulted in a considerable rise in research on the biosynthetic processes of aromatic terpenes in *Dendrobium* and other flowering plants. Nevertheless, the inquiry into the precise means by which plants regulate the proportion of diverse aromatic terpenes in their floral scent, thereby preserving their olfactory traits, requires further investigation. A conjecture on the botanical perfumer mechanism, which condensed the findings of earlier studies, was put forward to address this area of interest. Specific transcription factors likely govern the coordinated expression of multiple key terpene synthase (TPS) genes during the flowering stage of plants, thereby regulating the proportional biosynthesis of diverse aromatic terpenes and sustaining the distinctive aromatic properties of individual plants. This review serves as a significant theoretical reference for further investigations into aromatic volatile compounds in *Dendrobium*.

## 1. Introduction

Approximately 1500 species of *Dendrobium,* the second-largest genus in the Orchidaceae family, are found primarily in tropical Asia and Oceania [1,2]. Due to the abundance of alkaloidal, polysaccharide, and polyphenolic metabolites in the stems, species in the *Dendrobium* genus have been common ingredients in traditional Chinese medicine practices [3,4]. Meanwhile, a great amount of *Dendrobium* species hold high economic and ornamental value in the international flower trade [5]. Several *Dendrobium* species are popular in the ornamental flower market for their long blooming period, showy color, and strong fragrance [6]. The scent of ornamental flowers, known as the “soul” of the flower, is one of the most significant factors in the determination of its value [7]. According to several studies, the scent of the flower is a more influential factor than the color or the shape of the flower for consumers [8,9].

The term “floral scent” is used to describe volatile metabolites synthesized and released during the flowering cycle, which generally include several low-molecular-weight volatile organic compounds (VOCs) [10]. According to their biosynthesis processes and structural characteristics, floral VOCs are categorized as the following: terpenes, phenylpropanoids/ benzenoids, and fatty acid derivatives [10,11]. They are essential for attracting pollinators, defending them, and interacting with the environment. Moreover, floral VOCs are commonly employed in cosmetics, flavorings, fragrances, and even medicine [7]. More than 1700 floral VOCs have been found in 991 species of flowering plants and countless gymnosperms, with terpenes being most extensive class of secondary metabolites [10]. Due to its decorative and financial value, investigations on the floral scent of *Dendrobium* have increased in number in recent years [12]. Additionally, the dominant role of terpenes has steadily been shown in the formation of aromatic characteristics of *Dendrobium* [13]. Researchers are also starting to pay attention to several terpene biosynthesis-related genes in *Dendrobium* [14,15].

However, studies into the floral scent of *Dendrobium* have lagged considerably behind other important ornamental traits such as blossom shape and color [8]. One reason is that analogous research has historically been constrained by the accuracy of detection equipment to the simple identification of floral VOCs, and some results have been inconsistent [12]. Another point is that *Dendrobium* genome sequencing efforts were mostly directed towards species with medicinal properties [2]. Due to the aforementioned issues, accurate metabolic data and reference genomic information for further research are missing, and *Dendrobium* species’ genetic and molecular underpinnings of the floral scent are still little known. The detection of volatile aromatic terpenes, enzyme-encoding genes, and transcription factors involved in *Dendrobium* orchids’ life cycle are all discussed in detail in this review. We anticipate that our information will be useful in directing future research on *Dendrobium* floral scent.

## 2. Studies on Volatile Compounds of *Dendrobium*

### 2.1. History of Related Research and Landmark Findings

**Figure 1:**
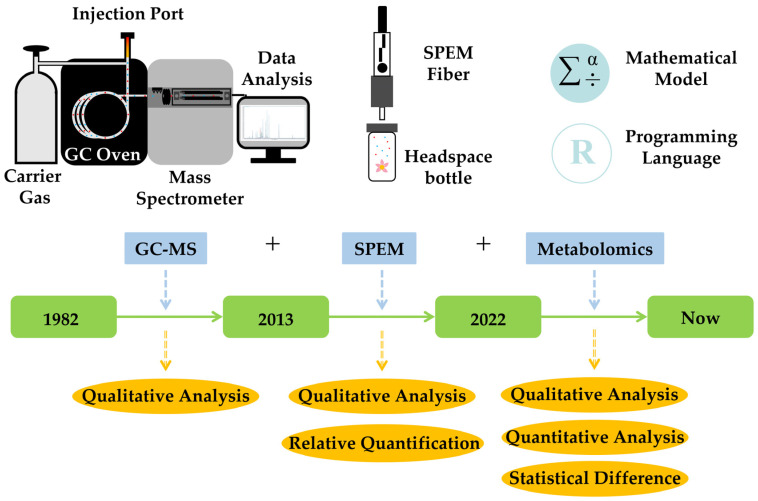
Stages of studies on volatile compounds of *Dendrobium*.

After a thorough assessment and organization of the studies on volatiles of *Dendrobium*, it became clear that the 1980s were the beginning of relevant studies in this area. (Figure 1; Table 1). Flath and Ohinata examined flowers of *D. superbum* (a synonym of *D. anosmum*) with gas chromatography–mass spectrometry (GC–MS), and 25 VOCs, including 2 terpenes, were detected [16]. Brodmann et al. investigated the floral scent correlated to pollinator attraction to *D. sinense*, and 5 VOCs were recognized in the samples of the floral organs [17]. Limited by the development level of detection technology, few numbers of components from floral fragrances could be identified at this stage, and only qualitative data were provided. As a result, the primary research goal at this stage was to search for pollinator-attracting chemicals among the limited number of floral VOCs.

To more fully characterize the volatile compounds derived from aromatic *Dendrobium*, the headspace solid-phase microextraction (HS-SPME) technique coupled with GC–MS was applied to the studies of floral fragrance. For the first time, 55 VOCs, including 12 terpenes, were isolated from *D. parishii* [18]. Subsequently, in sequence, researchers isolated and identified floral components from a variety of aromatic *Dendrobium* species using this technique, which is still widely used in the study of plant aromatic characteristics. The HS-SPME approach employed here is capable of accurately and sensitively extracting a plant’s VOCs without losing or destroying certain compounds in the process, resulting in a product that closely resembles the volatile chemicals found in the original plant. However, other traditional extraction techniques, such as steam distillation (SD), run the risk of losing or destroying chemicals during extraction and analysis [18]. Meanwhile, as the number of detections in floral components increased, some researchers attempted to quantify the detected volatile compounds. The peak area normalization approach (relation of each peak area to the total area) was used to determine the relative concentrations of volatile chemicals, in order to deliver the proportion of each component in the floral scent. In turn, they could determine which components had a more significant impact in the development of the floral characteristics of the samples. However, this quantitative analytical approach did have certain limitations. Firstly, it was not strictly accurate to define the total of the peak areas of the identified components as 100%, as we theoretically could not identify all components of the samples. Secondly, the data obtained by the peak area normalization method could not be statistically analyzed, so the investigators were unable to find statistically significant differential metabolites between samples. These shortcomings made the research results of this period stay at the level of simply identifying floral scent components.

With the continuous improvement and optimization of mass spectrometry, metabolite databases, and matching algorithms, HS-SPME GC–MS-based metabolomics technologies have emerged. Compared with previous methods, metabolomics has not only been improved in terms of qualitative analysis but also has fundamentally changed in terms of quantitative analysis. Following qualitative analysis of the samples, the metabolomic analysis process first combined and corrected the chromatographic peaks of the samples using internal standards, and then used mathematical models to compare the peak areas of the same compounds between the samples to filter out the key differential metabolites. In 2022, Wang et al. used this method and confirmed that terpenes, particularly monoterpenes, were primarily responsible for *D. loddigesii*’s fragrance production [12]. Immediately after, Du et al. detected more than 500 VOCs from *D. chrysotoxum* using an improved widely-targeted volatilomics method, which greatly improved the researchers’ knowledge about the complexity of *Dendrobium* floral scent. Furthermore, the results showed that terpenes have a significant influence on the floral characteristics of *D. chrysotoxum* [13]. Terpenes have so far been proven to have a major role in the development of the fragrance of *Dendrobium*. The study of *Dendrobium* fragrance has now officially advanced to a deeper level, thanks to the trustworthy metabolite data offered by metabolomics technology.

### 2.2. Aromatic Terpenes in the Floral VOCs of Dendrobium

**Figure 2:**
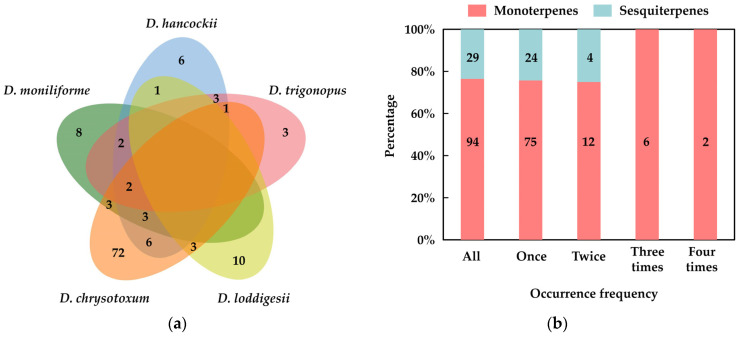
Aromatic terpenes of five aromatic *Dendrobium* species. (**a**) Venn diagram of terpenes in five *Dendrobium* species; (**b**) occurrence frequency of terpenes in five *Dendrobium* species.

Terpenes with isoprene structural units as their backbone are the most varied category of natural compounds among the various floral components. Mostly volatile monoterpenes (C10) and a few sesquiterpenes (C15) make up the terpenes in the floral scent. Diterpenes (C20), which have moderate volatility, are generally uncommon in floral compositions. Based on the data in published articles, we performed a statistical analysis of the terpene composition in floral fragrances of five representative aromatic *Dendrobium* species (Figure 2). The results suggested that the terpenes in *Dendrobium* floral fragrance consisted only of monoterpenes and sesquiterpenes, which aligned with the results of Kundsen et al. [10]. The monoterpenes with a high number of occurrences were cis/trans-ocimene (4), γ-terpinene (3), α-thujene (3), linalool (3), verbenone (3), myrcene (3), and limonene (3). In addition, the proportion of sesquiterpenes in shared terpenes gradually decreased as the number of chosen *Dendrobium* species increased. The results may imply that monoterpenes are essential in the formation of floral base notes in *Dendrobium*, while sesquiterpenes are mainly involved in the generation of aromatic differences.

## 3. Studies on Terpene Biosynthesis in *Dendrobium*

### 3.1. Terpene Biosynthetic Pathway

**Figure 3:**
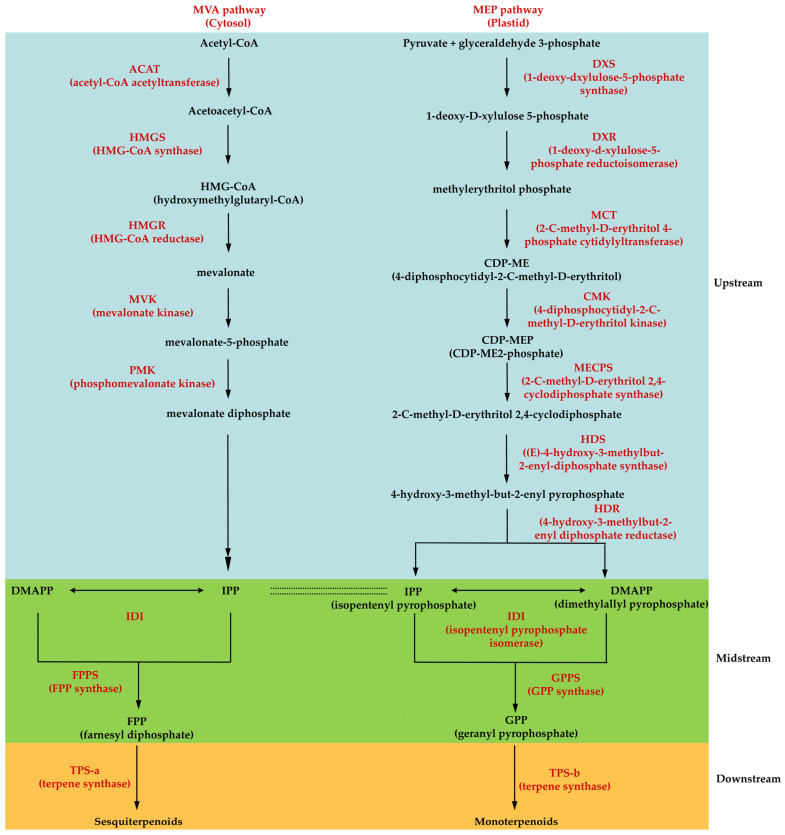
Terpene biosynthetic pathway of *Dendrobium*.

Currently, it is well understood how the upstream metabolic pathway, important metabolites, and genes involved in plant aromatic terpenes work (Figure 3). It consists of two relatively independent and linked pathways: the mevalonic acid (MVA) pathway located in the cytoplasm [27,28], and the methylerythritol phosphate (MEP) pathway located in the plastid [29]. Isopentenyl diphosphate (IPP) and dimethylallyl diphosphate (DMAPP), the substrates of terpenes produced by these two upstream pathways, are subjected to various isopentenyl transferases in the midstream to produce the monoterpene precursors geranyl diphosphate (GPP) and sesquiterpene precursors farnesyl diphosphate (FPP), respectively. These precursors are then directly catalyzed by various terpene synthases (TPSs) to produce diverse volatile aromatic terpenes, which constitute the dominant components of plant fragrance [11].

As the upstream pathways of terpene biosynthesis, MVA and MEP are not completely independent, but collaborate to provide IPP, the substrate for terpene production. HMGR and DXS are critical rate-limiting enzymes in the MVA and MEP pathways, respectively, affecting the supply of IPP(C5). Isopentenyltransferases, such as GPPS and FPPS, located midstream in the terpene synthesis pathway, are responsible for controlling the generation of precursors of various terpenes from IPP and its isomer DMAPP. In terpene biosynthesis, the main role of upstream and midstream enzymes is to regulate and provide precursors for the production of various terpenes, and their functions and coding sequences are highly conserved in most plants. In contrast, the quantity, type, and function of downstream TPSs vary considerably among species. Therefore, the diversity of terpenoids in plants depends directly on the diversity of TPSs’ species and activities [7]. In addition, many TPS family members can catalyze the production of multiple terpenes from a single substrate, which is another important factor in the formation of plant terpene diversity [11].

### 3.2. TPS in Dendrobium

Over 100 TPS genes have been screened and identified in model plants such as *Arabidopsis thaliana*, *Oryza sativa*, and *Solanum lycopersicum* [30,31,32]. Most plants have between 20 and 150 members of the TPS gene family, which consists of seven subfamilies (TPS-a, b, c, d, e/f, g, and h) based on the 40% homology in their amino acid sequences [33,34]. The TPS-a subfamily consists of sesquiterpene synthases and diterpene synthases in angiosperms. The monoterpene synthases that make up the TPS-b subfamily, all of which have been characterized, are also unique to angiosperms, and a conserved “R(R)X8W” motif can be found in the member’s amino acid sequence. Land plants contain the TPS-c subfamily, which differs from other subfamilies by the presence of the “DXDD” motif instead of the “DDXXD”. A subfamily exclusive to gymnosperms called TPS-d may encode mono-, sesqui-, and diterpene synthases. Most frequently found in vascular plants, the TPS-e/f subfamily encodes copalyl diphosphate synthases and kaurene synthases. The TPS-e/f subfamily was previously split into the TPS-e and TPS-f subfamilies separately, but further research revealed that TPS-f develops from TPS-e, leading to the joining of the two clades to form the TPS-e/f subfamily. Acyclic monocycles, sesquiterpenes, and diterpenes may be produced by members of the TPS-g subfamily, which are closely related to TPS-b but do not possess the conserved “R(R)X8W” motif. The “DXDD” and “DDXXD” motifs are shared by members of the TPS-h subfamily, which is currently restricted to *Selaginella moellendorffii* [34,35].

Studies related to TPSs in *Dendrobium* and other orchids have been reported. The *Dendrobium* TPSs are grouped into TPS-a, -b, -c, -e/f, and -g subfamilies, most of which belong to the TPS-a (64/168) and TPS-b (76/168) subfamilies (Table 2). Since monoterpenes dominate the aromatic terpene composition of *Dendrobium*, it is no surprise that TPS-b, which is responsible for monoterpene biosynthesis, accounted for the largest proportion of the *Dendrobium* TPS family members. In recent years, some researchers have started to focus on the functional identification of the TPS gene as a starting point for exploring the formation mechanism of aromatic characteristics in *Dendrobium*. Zhao et al. isolated the homologous gene of geraniol synthase *DoGES1*, a member of the TPS-b subfamily, from *D. officinale* by homologous cloning, and verified its function by prokaryotic expression and model plant transgenic assays [14]. Yu et al. identified the TPS gene family members of *D. officinale* and functionally characterized *DoTPS10* by prokaryotic expression, which showed that this TPS-b subfamily member is principally accountable for the biosynthesis of linalool [15]. Subsequently, Li et al. screened out 13 *DoTPS* genes associated with the accumulation of aromatic terpenes from *D. officinale* by a transcriptomic analysis of the two different cultivars [25].

### 3.3. Transcription Factors in Terpene Biosynthetic Pathway

In terms of transcriptional regulatory mechanisms, many genes involved in the terpenes synthesis pathway in plants exhibit some transcriptional correlation. This finding suggested that there should be some specific transcription factors in the terpene biosynthesis pathway, and that the expressions of related genes were likely to be regulated by the same class of transcription factors [39]. *GaWRK1* was the first transcription factor involved in the sesquiterpene biosynthesis pathway in cotton, followed by studies showing that *bHLH*-like transcription factors play a regulatory role in terpene biosynthesis in orchids such as *Phalaenopsis* and *Dendrobium* [40,41,42]. However, there were only a limited number of publications on transcription factors responsible for the control of terpene biosynthesis, and research in this field is still in the early phases of discovery and exploration.

## 4. Challenge and Opportunity

### 4.1. Current Dilemma: The Absence of Critical Scientific Issue in the Study of Floral Scent

There is a gap in the investigation of floral fragrances in *Dendrobium*. Researchers, such as breeders, have dedicated significant efforts towards elucidating the floral aroma components of various aromatic *Dendrobium* species. There may be some primary constituents in floral fragrances, but past research has shown that floral fragrances are made up of multiple compounds. However, their findings have been limited to the identification of basic floral fragrance components. While these studies have emphasized the prominent role of terpenes in shaping the aromatic characteristics of *Dendrobium*, they have not provided insight into the relationship between aromatic terpenes and the formation of these characteristics.

Some other researchers working on basic research realized the key role of TPS in the formation of aromatic terpene diversity and carried out a series of studies; the cloning and functional identification of TPS genes in *Dendrobium*. *D. officinale* has taken over as the preferred research material for this section of the study because of the better genomic information available. However, the floral fragrance of *D. officinale* was not prominent, making it an unsuitable species for research on the floral scent of *Dendrobium*. However, most of the metabolite data needed for this section of the study were missing, and the TPS genes under investigation were discovered by homology alignment. The products of these TPSs were also widely present in plant floral fragrance components, such as geraniol and linalool. These results revealed that specific TPS genes were involved in the development of the fragrance of *Dendrobium*, but researchers were unable to tell us if these genes were crucial or even major contributors to the development of the aromatic characteristics of *Dendrobium*.

The above-mentioned issues existed not only in the research field of *Dendrobium* but also widely in other aromatic plants. The researchers wanted to identify important genes that improve plants’ aromatic properties by resolving their aromatic characteristics, but it was possible that the genes they discovered and investigated did not fulfill this demand. This has led to an awkward situation in the field of plant aroma, in which some researchers only studied the floral components of plants while others focused only on the function of TPS genes. It is undeniable that the level of detection technology was an important factor contributing to this situation, but the absence of a critical scientific issue may be the root of the problem.

### 4.2. Presentation of Critical Scientific Issue and Conjecture on the Botanical Perfumer Mechanism

Why do we study floral scents? Aside from those researchers who concentrate on the connection between floral fragrance and pollinators, more researchers are attracted by the aromatic characteristics of plants, and thus recognize the high economic and research value of floral scents. Fragrance is not only one of the most important ornamental traits of flowers, but is also a key factor in helping people identify flower species. Most of the famous traditional flowers have unique aromatic characteristics, such as fresh jasmine, the charming rose, and the ethereal orchid. For most aromatic plants, their fragrance varies only in intensity between the different flowering stages, but their aromatic characteristics are fixed.

**Figure 4:**
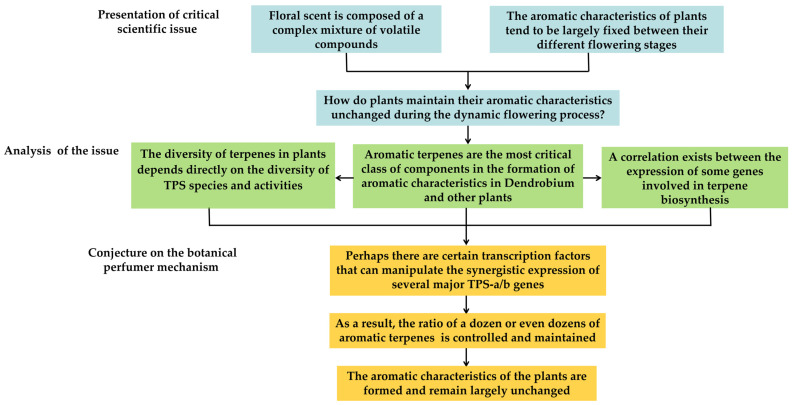
Presentation of critical scientific issue and conjecture on the botanical perfumer mechanism.

It is well known that the fragrant smell of flowers is composed of a complex mixture of volatile compounds that are normally emitted in a specific ratio. On the olfactory senses, it is as if there is a fixed “perfume formula” in each aromatic plant. Perfume blending is a delicate task, and a slight change in the ratio between volatiles is likely to change the scent of a perfume. The perfumer can control the ratio between different volatiles through droppers and measuring cups to keep the formula of a perfume constant. How do plants perform this complex and delicate work in the dynamic processes of blossoming and fragrance emission? We believe that this is the key issue in the research on floral scent (Figure 4). There should be a specialized regulatory mechanism in the plants to maintain their aromatic characteristics during the dynamic flowering process, which we call the botanical perfumer mechanism.

Once the key question is derived, previous research could be systematically organized around it and transformed into a scientific issue. Aromatic terpenes are a dominant factor in the development of aromatic characteristics in *Dendrobium*, and the diversity of aromatic terpenes in plants depends largely on the type and activity of TPS-a/b. It is not difficult to deduce that TPS-a/b should occupy a central position in the botanical perfumer mechanism. Meanwhile, a correlation exists between the expression of some genes involved in terpene biosynthesis. Moreover, based on the property of TPS as a multiproduct enzyme, a plant could likely control the ratio of a dozen or even dozens of aromatic terpenes by manipulating the synergistic expression of only several TPS-a/b members in its body; this is already sufficient to form its aromatic characteristics.

### 4.3. An Example of Related Research on D. chrysotoxum

**Figure 5:**
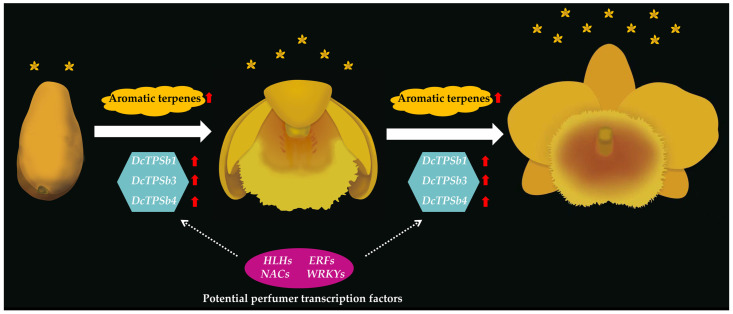
A potential example of the botanical perfumer mechanism in *D. chrysotoxum* [13]. The red arrows represent the tendency of up-regulation; the number of orange florets characterize the intensity of aromatic characteristics.

An example similar to the above conjecture has been observed in experiments (Figure 5). The characteristics and biosynthetic mechanism of floral fragrance in *D. chrysotoxum* during blooming were uncovered using widely-targeted volatilomics in conjunction with transcriptome analysis. The results of the transcriptomics and volatilomics studies revealed that terpenes and associated genes are crucial in the development of aromatic characteristics in *D. chrysotoxum*. Surprisingly, across various blooming phases, the expression of the majority of upstream structural genes implicated in the MEP and MVA pathways was continuous and stable. Downstream TPS-b genes, on the other hand, tended to exhibit an up-regulated pattern with blooming, which aligned with the accumulation of volatile terpenes in floral organs.

Subsequently, an aromatic terpene transcriptional and metabolic regulatory network centered on three TPS genes was constructed by joint analysis of transcriptional and metabolic data. The network linked three TPS-b genes to 26 representative aromatic terpenes and 21 transcription factors. The expressions of the three TPS-b genes were consistently up-regulated, along with the flowering process of *D. chrysotoxum*, and maintained a high expression level, which showed a strong correlation with the accumulation of many representative aromatic monoterpenes in the floral scents of *D. chrysotoxum*. This suggested that these three TPS genes are likely to be the major genes for the biosynthesis of aromatic terpenes in *D. chrysotoxum*. Notably, the three *DcTPSs* genes showed a high correlation at the transcriptional level, suggesting that the expression of the major *DcTPSs* genes is likely to be regulated by some or even the same class of transcription factors during the biosynthesis of aromatic terpenes. Furthermore, it is likely that these unknown transcription factors are the “perfumers” of the plants we long to find.

## 5. Conclusions

In the past decades, there has been significant progress in the identification of aromatic terpenes and functional studies of TPS genes in *Dendrobium*. However, the wide variety of aromatic terpenes and TPS gene family members have left researchers with a lack of overall knowledge about the metabolic regulation of aromatic terpenes. The conjecture on the botanical perfumer mechanism provides an idea to improve this situation. Screening for major TPS genes and searching for transcription factors that regulate their synergistic expression is a meritable research focus for the floral scent of *Dendrobium* and other plants. This will help enrich the theory related to the metabolic regulation of aromatic terpenes, and lay the foundation for finding the key factors that regulate the metabolism of plant aromatic terpenes.

## Figures and Tables

**Table 1:** Representative study of volatiles of *Dendrobium*.

Species	Publication Time	Material	Method	VOCs	Terpenes
*D. superbum*	**1982** [16]	Flowers	GC–MS	25	2
*D. sinense*	2009 [17]	Flowers	GC–MS	5	0
*D. parishii*	**2013** [18]	Flowers Leaves Roots	HS-SPEM GC–MS	55	11
*D. nobile*	2015 [19]	Flowers	DHS GC–MS	32	4
*D. moschatum*	2018 [20]	Flowers Leaves	SD GC–MS/GC–FID	46	1
*D. chrysotoxum*	2018 [21]	Flowers	HS-SPEM GC–MS	33	17
*D. chrysotoxum* *D. chrysanthum* *D. harveyanum* *D. wardianum* *D. amabile*	2021 [6]	Flowers	SD GC–MS/GC–FID	93	16
*D. nobile*	2019 [22]	Flowers	HS-SPEM/SD GC–MS	69	21
*D. moniliforme*	2019 [23]	Flowers	HS-SPEM GC–MS	59	18
*D. hancockii* *D. trigonopus*	2020 [24]	Flowers	HS-SPEM GC–MS	72	27
*D. officinale*	2021 [25]	Flowers	HS-SPEM GC–MS	13	13
*D. loddigesii*	**2022** [12]	Flowers	Non-target metabolomics	80	14
*D. officinale*	2022 [26]	Flowers	HS-SPEM GC–MS	34	9
*D. chrysotoxum*	**2022** [13]	Flowers	Widely-targeted volatilomics	543	90

Bolded years represent landmark studies; DHS: dynamic headspace; SD: steam distillation; GC–FID: gas chromatography–flame ionization detector.

**Table 2:** The amount of TPS subfamilies in *Dendrobium* and other orchids.

Species	a	b	c	e/f	g	Total	Reference
*Apostasia shenzhenica*	2	4	0	1	2	9	[36]
*Phalaenopsis equestris*	4	7	0	4	0	15	[36]
*P. aphrodite*	6	7	0	4	0	17	[36]
*P. bellina*	1	7	0	3	0	11	[36]
*Dendrobium catenatum*	13	18	0	4	0	35	[36]
*D. officinale*	14	16	1	3	0	34	[15]
*D. chrysotoxum*	16	23	2	7	0	48	[37]
*D. nobile*	21	19	5	6	0	51	[38]

## Data Availability

Not applicable.
